# Is Working Longer the Solution to an Aging Society?

**DOI:** 10.1093/geroni/igaa057.2937

**Published:** 2020-12-16

**Authors:** Phyllis Moen

**Affiliations:** University of Minnesota, Minneapolis, Minnesota, United States

## Abstract

Population aging, together with increases in life expectancy, women’s employment, dual-earner households, and unraveling safety nets, are transforming the demography of work and retirement in the later life course. A seemingly obvious policy solution is to encourage Boomers and those following to work longer, postponing retirementt. And yet there is insufficient understanding of factors that predict ongoing older adult participation in paid work. The simple work/retirement dichotomy no longer fits the experience of growing numbers of Americans. Drawing on census data, I show six such pathways differing by age, gender, and race/ethnicity. Needed is policy development recognizing the new realities of different and unequal pathways. The challenge is recognizing disparities in capacities and constraints among our aging population, along with the need to change the way we work, opening up flexible options in order to enable working longer.

